# The Disaster of the Century: Effects of the 6 February 2023 Kahramanmaraş Earthquakes on the Sleep and Mental Health of Healthcare Workers

**DOI:** 10.3390/ijerph21111533

**Published:** 2024-11-19

**Authors:** Sema Çifçi, Zehra Kilinç

**Affiliations:** 1 Nursing Department, Faculty of Health Sciences, Mardin Artuklu University, Mardin 47100, Türkiye; 2 Department of Public Health, Faculty of Medicine, Dicle University, Diyarbakır 21280, Türkiye; drzehrakln@hotmail.com

**Keywords:** earthquake, health workers, mental health, sleeping

## Abstract

It is known that disasters can have long-term effects on the mental health of individuals. In particular, healthcare workers may be under greater stress in a time of disaster, as they are not only affected by the disaster, but they also take part in rescue efforts. This study was conducted to investigate the psychological effects of the Kahramanmaraş earthquakes on healthcare workers employed at the Adıyaman Training and Research Hospital. The sample in the cross-sectional study consisted of 299 healthcare personnel working in the Adıyaman Training and Research Hospital. The study data were collected through a questionnaire consisting of five sections. The data were analysed using SPSS 22 software. It was found that among healthcare workers, those who were women, married, individuals whose homes were damaged, injured, or lost a relative in the earthquake had experienced mental health problems such as post-traumatic stress disorder (PTSD), anxiety, depression, and poor sleep quality the most. The earthquakes that occurred on 6 February negatively affected the mental health of healthcare workers. In order to reduce these negative effects experienced by healthcare workers, various types of mental health screening should be performed, and supportive psychological services should be provided urgently.

## 1. Introduction

Disasters are events that cause serious disruptions to the functioning of a society, causing it to exceed its capacity to cope using its own resources [1]. The effects of disasters can endure long after their occurrence. Studies have shown that the mental health of disaster survivors may be profoundly and permanently affected [2,3]. Earthquakes are among the most common and devastating natural disasters. Throughout history, earthquakes in various parts of the world have not only resulted in the deaths of hundreds of thousands of people and injuries to millions, but they have also led survivors to experience various traumas [4]. Individuals who have had to wait under the rubble of collapsed buildings for rescue, who have lost loved ones and family members, or whose homes have been destroyed may experience a range of mental health problems, including post-traumatic stress disorder (PTSD), depression, and anxiety [5,6]. PTSD and depression significantly disrupt the social lives of those affected by disasters [7,8]. Individuals who have experienced earthquakes are at risk of developing PTSD, anxiety, depression, and insomnia. Studies indicate that nearly a quarter of individuals who survive major earthquakes are diagnosed with PTSD [9]. Sleep disorders are among the most common health problems encountered after traumatic events [10]. Research shows that sleep patterns are disrupted following such events, and these disorders can exacerbate the development of depression and anxiety [11,12]. Individuals affected by disasters often experience various sleep problems, including difficulty falling asleep, frequent awakening, short sleep duration, restless sleep, daytime fatigue associated with sleep disorders, respiratory issues, and nightmares [3]. Among those who have survived earthquakes, the prevalence of sleep disorders has been reported to range from 10.7% to 83.2%. These disorders may develop immediately after an earthquake and can persist for extended periods, from 8 months to 15 years [10]. Long-term studies indicate high rates of depression among disaster survivors, even years after the event. Traumatic deaths within the family, coupled with a lack of necessary social support, increase the risk of depression. In communities affected by disasters, addressing the psychological problems that may arise is of vital importance for public health [13,14,15]. Recognizing trauma-related disorders, such as post-earthquake sleep disorders, PTSD, anxiety, and depression, and implementing timely and appropriate interventions will contribute to reducing long-term mental health problems [16].

Earthquakes are traumatic events that can have significant psychological effects on individuals. The fear, stress, and anxiety associated with earthquakes may lead to various mental health issues, such as post-traumatic stress disorder (PTSD), anxiety disorders, and depression. These psychological conditions can severely disrupt sleep and contribute to the emergence of sleep disorders [17].

During disasters, healthcare workers providing emergency medical services may encounter horrifying scenes, including dead bodies, severely injured individuals, and distressed patients. Consequently, healthcare professionals are vulnerable to occupational stressors, which can adversely affect their mental health. These effects can manifest as PTSD, depression, and other psychological disorders [18,19].

A systematic review has found that the prevalence of PTSD among healthcare workers following earthquakes ranges from 3.2% to 30%. In the same study, the prevalence of depression was reported to be 27.1% [20].

As a result of the 7.7 and 7.6 magnitude earthquakes that occurred in the Pazarcık and Elbistan districts of Kahramanmaraş on 6 February 2023, a state of emergency was declared in the provinces of Kahramanmaraş, Hatay, Adıyaman, Malatya, Kilis, Gaziantep, Diyarbakır, Şanlıurfa, Osmaniye, Adana, and Elazığ. According to official data, 50,783 people were killed and 115,353 were injured in the earthquakes. Additionally, it was reported that 37,984 buildings were destroyed [21]. The primary focus of studies conducted on the effects of these earthquakes has been on the civilian population affected by the disaster [3,7,9,10,11,16]. In contrast, there are a limited number of studies addressing healthcare workers, who are both victims of disasters and directly involved in disaster response efforts; thus, they are affected in multiple ways. Healthcare workers directly engaged in disaster settings experience increased stress due to heightened workloads. Therefore, it is crucial to evaluate the mental health of healthcare workers, who play a vital role in providing healthcare services in both the short- and long-term following an earthquake. The sustainability and quality of healthcare services depend on safeguarding the mental health of these professionals. In this context, further research on the mental health of healthcare workers—specifically regarding PTSD, anxiety, depression, and sleep disorders—will contribute to the development of strategic interventions tailored to their needs.

This study aims to investigate the psychological effects of the 6 February 2023, Kahramanmaraş earthquakes on healthcare workers in Adıyaman, particularly focusing on post-traumatic stress disorder (PTSD), anxiety, depression, and sleep disorders.

## 2. Materials and Methods

### 2.1. Study Design

This study was designed as cross-sectional research. It aims to assess post-traumatic stress disorder (PTSD), anxiety, depression, and sleep quality among healthcare workers in Adıyaman following the Kahramanmaraş earthquakes. Based on the findings, the study seeks to contribute to the development of mental health interventions tailored for this group.

### 2.2. Participants/Sample

The population of the study comprised 3014 healthcare workers employed at the Adıyaman Training and Research Hospital. The sample of the study was determined as at least 341 people according to calculations made using the OpenEpi 3.0.1 software with a 95% confidence interval and a 5% margin of error [22]. At the end of the study, it was possible to include 299 people. The reasons that the entire target population could not be reached include intense working conditions in the hospital, mental and physical fatigue after the earthquakes, and lack of voluntary participation.

### 2.3. Inclusion Criteria

Working as medical personnel in any unit of the Adıyaman Training and Research Hospital.

### 2.4. Exclusion Criteria

Being pregnant or in the postpartum period at the time the study was being conducted.

Not agreeing to participate in the study willingly or voluntarily.

### 2.5. Limitations of the Study

The study population is limited to healthcare workers at the Adıyaman Training and Research Hospital. Although this study was conducted in a single institution, its findings may provide valuable insights into the mental health issues and sleep disorders experienced by healthcare workers in the aftermath of a disaster.

The fact that the research is based on voluntary participation carries the risk of volunteer bias. Healthcare workers who are more sensitive about mental health may be more likely to have participated in the study.

The study was conducted over a short period of time. The data were collected approximately one year after the earthquakes. More time and further research are needed to reveal the long-term effects of the earthquakes on the mental health of individuals.

The present study has limitations, including the omission of factors such as family size. This aspect should be taken into account in future research. It is recommended that this factor be considered in subsequent studies.

### 2.6. Data Collection Tools

A survey, which comprised of five sections, was used to collect the study data: In Section 1, questions to determine the socio-demographic characteristics of the participants (age, gender, marital status, professional seniority, parental status) were included [3,4,9,10].

In Section 2, the Post-Traumatic Stress Disorder (PTSD) Short Scale, developed by LeBeau et al. (2014) and adapted into Turkish by Evren et al. (2016), was used. The scale is a Likert-type scale that consists of nine items, in which higher scores indicate increased severity of PTSD. The cut-off value for the scale was determined as 24. The value of Cronbach’s alpha was found to be 0.88 in the study [23,24].

In Section 3, the Beck Anxiety Inventory (BAI), developed by Beck et al. (1988) and adapted into Turkish by Ulusoy et al. (1998), was used. According to studies conducted by Ulusoy et al. (1998) on the validity and reliability of the inventory, the Cronbach’s alpha reliability coefficient was found to be 0.92. The scale is a Likert-type scale that consists of 21 items and each item is scored from 0 to 3. The highest score that can be obtained is 63. A score between 8 and 15 is classified as ‘mild anxiety’, 16–25 as ‘moderate anxiety’, and 26–63 as ‘severe anxiety’ [25,26].

In Section 4, the Beck Depression Inventory (BDI), a 21-item self-rated scale developed by Beck (1961), was used. Each item in the BDI is scored from 0 to 3. The total score to be obtained from the scale ranges from 0 to 63. A score above the determined cut-off point for the BDI, which is 17, indicates the presence of depression. A score between 0 and 9 is classified as “normal”, 10–18 as “mild depression”, 19–29 as “moderate depression”, and 30–63 as “severe depression” [27].

In Section 5, the Pittsburgh Sleep Quality Index (PSQI), which was developed by Buysse et al. (1989) and adapted into Turkish by Ağargün et al. (1996), was used. The PSQI consists of 24 items in total, 19 of which are self-rated questions for participants. Factors affecting sleep quality are evaluated in seven subcomponents: “subjective sleep quality”, “sleep latency”, “sleep duration”, “sleep efficiency”, “sleep disturbances”, “sleeping medication use” and “daytime dysfunction”. The index has a total score that ranges from 0 to 21, and a score above 5 indicates poor sleep quality. Higher scores show that sleep quality has decreased [28].

### 2.7. Data Collection Process

Study data were collected between 15 December 2023 and 15 January 2024, by conducting a face-to-face survey with healthcare workers employed at the Adıyaman Training and Research Hospital who agreed to participate in the study. Written and verbal consent were obtained from the participants, who had been informed about the research.

The data collection process was conducted by the researchers. To ensure the consistency of the interviewers’ assessments, a half-day training program was implemented prior to data collection. The interviews were conducted in accordance with a pre-prepared protocol.

In this study, a pilot application was conducted with 10 healthcare workers to assess the validity of the demographic data and the appropriateness of the interview process. During this pilot study, the participants’ comprehension of the questions and their response duration were evaluated. Based on the findings, minor adjustments were made to the interview process and some question formulations. These improvements aimed to enhance the efficiency of the main study and optimize the data collection process. The data obtained from the healthcare workers who participated in the pilot study were not included in the analysis phase.

### 2.8. Statistical Analysis

Data were analyzed using the SPSS (Statistical Package for Social Sciences; SPSS Inc., Chicago, IL, USA) 22 software package. In the study, descriptive data were expressed as numbers and percentages for categorical variables, and as mean ± standard deviation (Mean ± SD) or median and interquartile range (the 25th and 75th percentiles) for continuous variables. A chi-square analysis (Pearson’s chi-square test) was used to compare categorical variables between the groups. The suitability of continuous variables for normal distribution was evaluated with the Kolmogorov–Smirnov test. The Mann–Whitney U-test was used in the comparison of two groups, and the Kruskal–Wallis test was used in the comparison of more than two variables. The Spearman’s rank correlation test was used to examine the relationship between continuous variables. A linear regression analysis was performed to determine the predictors of the scale scores. The enter method was used when building the model, and those variables with a significant relationship detected in the correlation test and pairwise comparisons were included in the model. In the analyses, a *p* value of <0.05 was considered statistically significant.

### 2.9. Ethical Considerations of the Study

Prior to the study, necessary legal permissions were obtained from the Non-interventional Clinical Research Ethics Committee of Mardin Artuklu University (06.11.2023/11) and the Adıyaman Provincial Directorate of Health. The study was conducted in accordance with the Declaration of Helsinki. Informed consent was obtained from the participants. The data were stored in a secure environment and used for scientific purposes only. The principles of confidentiality and anonymity were followed throughout the study. 

## 3. Results

In the study, 195 (65.2%) of the participants were female and 104 (34.8%) were male. The mean age of the participants was found to be 33.8 ± 8.7 (min = 17; max = 58) years. Of the healthcare workers included in the study, 68.6% had a bachelor’s degree, 6.4% had a master’s/doctoral degree, and 58.5% were married. A total of 6.7% of the participants were doctors, 62.2% were nurses/midwives, 17.4% were technicians, 9.7% were medical secretaries, and 4.0% were in the “other” occupational group. Of the participants, 94.0% had experienced the earthquakes. The homes of 85.3% of the healthcare workers were damaged and 55.2% of the participants were living in temporary housing. Relatives of 79.3% of the participants lost their lives in the earthquakes and 16.7% of the participants were injured. Moderate levels of anxiety, depression, and post-traumatic stress disorder symptoms were observed in the participants. It was determined that they had poor sleep quality overall (Table 1).

The Beck Anxiety Inventory (BAI), Beck Depression Inventory (BDI), Post-Traumatic Stress Disorder (PTSD) Short Scale, and Pittsburgh Sleep Quality Index (PSQI) scores of women were found to be significantly higher than those of men (*p* < 0.001). Similarly, the anxiety, PTSD, and sleep quality scores of married participants were significantly higher than those of single participants (*p* < 0.001, *p* = 0.011, *p* = 0.018). The BAI, PTSD and PSQI scores of doctors were found to be significantly lower than those of nurses, midwives, technicians, and medical secretaries (*p* = 0.002, *p* = 0.007, *p* = 0.049). The PTSD scores of participants who experienced the earthquakes were significantly higher than the scores of those who did not experience the earthquakes (*p* = 0.019). In addition, it was observed that the BDI scores of participants working at the hospital during the earthquake were higher than the scores of those who were at home (*p* = 0.040). The BAI, BDI, PTSD, and PSQI scores of participants whose homes were damaged were significantly higher (*p* < 0.01). Similarly, it was observed that participants who were injured in the earthquake and those who lost relatives had higher scores on mental health scales (*p* < 0.05) (Table 2).

According to the correlation analyses conducted, a significant positive correlation was identified between the Beck Anxiety Inventory (BAI) score and the Beck Depression Inventory (BDI), Post-Traumatic Stress Disorder (PTSD) symptoms, Pittsburgh Sleep Quality Index (PSQI) scores, age, and years of professional experience. This finding indicates that as anxiety levels increase, the incidence of depression, post-traumatic stress, and sleep disorders also rises. Additionally, both age and years of professional experience are associated with anxiety levels. Conversely, a significant negative relationship was observed between the BAI score and educational status, suggesting that anxiety levels decrease as educational level increases.

A significant positive relationship was detected between the Beck Depression Inventory (BDI) score and scores on both the Post-Traumatic Stress Disorder (PTSD) Short Scale and the Pittsburgh Sleep Quality Index (PSQI), as well as weekly working hours. As the level of depression increased, instances of post-traumatic stress also rose, and sleep quality deteriorated; furthermore, higher levels of depression were observed with increased weekly working hours. In addition, a positive correlation was found between PTSD scores, PSQI scores, and the number of years in the profession, indicating that sleep quality is impaired in individuals experiencing post-traumatic stress. Those with longer tenure in the profession are at a greater risk for developing PTSD. Additionally, a significant negative relationship was identified between PTSD and educational status, suggesting that the risk of PTSD decreases with higher levels of education. Significant positive correlations were also observed between PSQI scores and age, years in the profession, and the extent of damage to homes, indicating that sleep quality worsens with advancing age and longer professional experience. Moreover, participants whose homes were damaged reported poorer sleep quality. A negative correlation was detected between PSQI scores and income status, revealing that individuals with higher income levels experienced better sleep quality (Table 3).

According to the multiple linear regression analysis, BAI scores were predicted by BDI score (β = 0.263, *p* < 0.001), PTSD score (β = 0.928, *p* < 0.001), age (β = 0.356, *p* = 0.010), educational status (β = −20.010, *p* = 0.007), and occupation (β = −20.428, *p* < 0.001). BDI scores were predicted by BAI score (β = 0.238, *p* < 0.001), PSQI score (β = 0.565, *p* = 0.003), and the injury status in the earthquake (β = −30.419, *p* = 0.025). PTSD scores were predicted by BAI score (β = 0.325, *p* < 0.001), PSQI score (β = 0.598, *p* < 0.001), gender (β = −20.262, *p* = 0.003), occupation (β = 10.488, *p* < 0.001), presence of injured family members (β = −10.849, *p* = 0.022), and loss of relatives in the earthquake (β = −20.766, *p* = 0.002). PSQI scores were predicted by BDI score (β = 0.047, *p* = 0.022) and PTSD score (β = 0.132, *p* < 0.001) (Table 4).

## 4. Discussion

This study was intended to comprehensively examine the effects of major natural disasters such as earthquakes on the mental health and sleep quality of healthcare workers. It was found that healthcare workers generally experienced moderate levels of post-traumatic stress disorder (PTSD), anxiety, depression, and poor sleep quality. Studies show that similar events cause various psychological effects on healthcare workers. It has been observed that mental health problems, especially PTSD, anxiety, and depression increase significantly in post-disaster periods [18,29].

In the present study, it was found that women had higher PTSD scores than men (*p* < 0.001), and nurses had higher PTSD scores than doctors (*p* = 0.026). In various studies, it has been reported that trauma-related responses among female healthcare workers indicate higher levels of stress and trauma symptoms in comparison with their male counterparts [30,31]. In a meta-analysis conducted in 2023, it was observed that PTSD scores were higher in certain groups of healthcare workers involved in the post-earthquake response [18]. The meta-analysis results indicated that the prevalence of post-earthquake PTSD among medical workers involved in the earthquake response was 16.37%.

In a study by Mealer et al. (2009), it was shown that nurses were at greater risk of PTSD due to increased workload and involvement in direct patient care [32]. In a study conducted by Keya et al. (2023), differences in stress and trauma levels between doctors and other healthcare personnel were examined, and it was suggested that these differences arose due to professional roles. The educational attainment of healthcare workers, the unit they work in, the roles they undertake and the time they spend with patients differ from one another. Therefore, mental health problems that may arise after a disaster are likely to vary in terms of severity [33,34,35].

In the present study, it was determined that married healthcare workers had higher PTSD scores than single healthcare workers. In the study of Carmassi et al. (2022), it was stated that married healthcare workers were found to experience different levels of stress and trauma than single healthcare workers [33]. In our study, it was found that participants who were married, whose homes were damaged, who were injured, or lost their relatives had higher PTSD scores. In similar studies, it has been shown that participants who were married and had children had higher PTSD scores [36,37]. Our findings are consistent with the literature. It can be said that the roles and responsibilities undertaken by individuals, anxiety, and experience of losing loved ones have an impact on the development of PTSD.

In the present study, female and married participants had higher anxiety scores than male and single participants. In another study by Liu et al. it was also shown that female healthcare workers experienced anxiety more than male healthcare workers [38]. In a study by Chen et al. (2020), it was stated that married healthcare workers had higher levels of anxiety compared to their single colleagues due to marital responsibilities [39]. It has also been reported in the literature that participants whose homes were damaged by an earthquake had higher anxiety levels. In the study of Dai et al. (2016), it was emphasized that damage to homes undermined the sense of safety and increased anxiety [9]. In a similar study, it was found that grief and trauma have negative effects on the mental health of healthcare workers who have lost loved ones [40].

Married participants had poorer sleep quality than single participants. This suggests that family responsibilities and fear of losing loved ones may have negatively affected sleep quality. In participants whose homes were damaged, higher PSQI scores were detected. In a study by Dai et al. (2016), sleep disorders were found to have increased due to the damaged sense of safety after trauma. In our study, it was determined that doctors had lower PSQI scores compared to other healthcare workers. The status and roles of doctors may have had an impact on their PSQI scores [9].

Participants who were working at the hospital during the earthquakes had higher Beck Depression Inventory (BDI) scores than those who were at home. This shows that intense working conditions in the hospital and having been involved in direct response to traumatic events may increase the risk of depression. It was observed that individuals, whose homes were damaged also had higher BDI scores. Our findings are consistent with the literature These findings show that environmental and personal factors experienced during and after earthquakes have an impact on depression levels in individuals [40,41,42].

Natural disasters, particularly earthquakes, have significant psychological effects on healthcare workers, as well as on all segments of society. The negative effects of physical losses and trauma on mental health are frequently emphasized in the literature. The high levels of depression and anxiety detected in individuals who were injured or lost their relatives in the earthquakes indicate that these individuals experienced greater psychological distress in the face of trauma. In a systematic review conducted by Bohlken et al., it was determined that injuries to individuals and family members negatively affected mental health. There were negative effects of grief and trauma on the mental health of healthcare workers who lost their loved ones. In similar studies, it has been shown that the mental health of individuals, especially those who lost loved ones or were injured in traumatic events, was negatively affected [40]. Natural disasters such as earthquakes cause unexpected changes in the lives of individuals due to their sudden and destructive effects. It may be difficult and time-consuming for individuals to get used to this new situation in terms of mental health [43,44].

In this study, significant positive relationships were detected between mental health problems such as PTSD, anxiety, and depression (β = 0.928, *p* < 0.001). In a study by Maslach and Leiter (2016), burnout was reported to be associated with other psychological disorders such as depression, anxiety, and PTSD, which corroborates the findings from our study. Our findings show that sleep quality is impaired in individuals suffering from post-traumatic stress disorder and that those who have worked in the profession for a long time are at greater risk of PTSD [45]. The finding of a study conducted by Kamalulil et al. that the number of years in the profession may increase the risk of PTSD confirms our findings (β = 0.598, *p* < 0.001). In our study, a negative relationship was found between income status and PSQI (β = −20.010, *p* = 0.007). In a study by Naushad et al., the effects of socioeconomic status on mental health were examined and mental health problems were found to be more common in individuals with low levels of income. The findings of the present study overlap with those in the literature. For healthcare professionals, having to work for a long time and at an intense pace in the same professional field can be physically and mentally exhausting over time. In our study, a positive relationship was found between age and BAI scores (β = 0.356, *p* = 0.010). These findings show that the severity of anxiety increases with age [46,47].

A positive relationship was found between weekly working hours and PTSD, BDI, and PSQI. As the level of depression increased, post-traumatic stress also increased and sleep quality deteriorated; furthermore, higher levels of depression were observed as the weekly working hours increased. In a study by Dollard and Bakker (2010), the negative effects of long working hours on mental health were discussed. These findings indicate that the factors affecting the trauma-related responses of healthcare workers are multifaceted. Natural disasters, in addition to the traumatic effects they have on individuals, cause PTSD and deterioration in sleep quality due to suddenly increased workload [48,49].

## 5. Conclusions

This study shows that after the 6 February 2023 Kahramanmaraş earthquakes, negative effects have occurred on the mental health of healthcare professionals working in the disaster area. Female healthcare workers, those who are married, who have been injured, or who lost loved ones in the earthquakes are at greater risk for mental health problems such as post-traumatic stress disorder (PTSD), anxiety, depression, and poor sleep quality. The present study focuses on the mental health issues and sleep disorders of healthcare workers following the earthquakes. However, considering the similarities in experiences among healthcare workers in disaster areas, it can be inferred that similar mental health issues may also apply to other disasters such as pandemics, floods, and fires. The responsibilities undertaken by healthcare workers—including medical intervention, patient care, and administrative management—create a significant physical and psychological burden, thereby increasing the risk of anxiety, depression, post-traumatic stress disorder (PTSD), and sleep disorders. Direct involvement in patient care or disaster management is associated with an escalation in these mental health issues. These findings provide important insights for better understanding the effects of working conditions on healthcare workers’ mental health and for developing interventions to mitigate these effects.

When their roles and responsibilities in times of disaster are taken into consideration, it will be better understood that protecting the mental health of healthcare workers after disasters is of vital importance for the sustainability of healthcare services.

The findings of the present study also indicate that governments should take greater responsibility for supporting the mental health of their employees during and after major disasters. In this context, several practical steps can be recommended for healthcare institutions:

- Mental health professionals should provide regular screenings and confidential counseling services for healthcare workers, which can help alleviate the psychological burden in disaster environments.
- Care models should be developed to protect the mental health of healthcare workers after a disaster. It is important to develop more comprehensive and continuous psychosocial support programs, especially for healthcare professionals working in disaster areas. This process should be supported by creating long-term rehabilitation and monitoring programs.
- Incorporating stress management and resilience training into disaster preparedness programs can enhance healthcare workers’ coping skills for traumatic events.
- Long-term monitoring of the mental health of healthcare workers post-disaster allows for the identification and intervention of delayed or cumulative trauma effects.
- To support the morale and motivation of healthcare workers engaged in disaster response, short-term additional leave can be granted. It is suggested that these leave periods be spent in stress-free locations outside the disaster area, with financial and emotional support provided for this purpose.

## Figures and Tables

**Table 1:** All characteristics of the participants.

		n	%
**Gender**	Female	195	65.2
	Male	104	34.8
**Age Median (IQR)**		32.0 (27.0–40.0)	
**Educational status**	High school degree	15	5.0
	Associate’s degree	60	20.1
	Bachelor’s degree	205	68.6
	Master’s/doctoral degree	19	6.4
**Marital status**	Married	175	58.5
	Single	124	41.5
**Duration of marriage, Median (IQR)**		10.0 (4.5–17.0)	
**Number of years in the profession, Median (IQR)**		9.0 (3.0–16.0)	
**Weekly working hours**	≤40 h	178	59.5
	41–50 h	103	34.4
	>50 h	18	6.0
**Income status**	Income is less than expenditure	146	48.8
	Income is equal to expenditure	116	38.8
	Income exceeds expenditure	37	12.4
**Occupation**	Doctor	20	6.7
	Nurse/midwife	186	62.2
	Technician	52	17.4
	Medical secretary	29	9.7
	Other	12	4.0
**Status of having experienced the earthquakes**	Yes	281	94.0
	No	18	6.0
**Location during the earthquakes**	At home	264	94.0
	At the hospital	17	6.0
**Presence of damage to home**	Yes	255	85.3
	No	44	14.7
**Level of damage to home**	Mild damage	112	43.9
	Moderate damage	51	20.0
	Severe damage	62	24.3
	Collapsed	30	11.8
**Current place of residence**	Own house	134	44.8
	Temporary housing	165	55.2
**Injury status in the earthquake**	Yes	50	16.7
	No	249	83.3
**Presence of injured family members**	Yes	80	26.8
	No	219	73.2
**Loss of relatives in the earthquake**	Yes	237	79.3
	No	62	20.7
**BAI, Median (IQR)**		19.0 (9.0–30.0)	
**BDI, Median (IQR)**		16,0 (10.0–23.0)	
**PTSD, Median (IQR)**		18.0 (11.0–25.0)	
**PSQI, Median (IQR)**		9.0 (7.0–12.0)	

**Table 2:** Comparison of scale scores according to various parameters.

		BAI	BDI	PTSD	PSQI
		Median (IQR)	Median (IQR)	Median (IQR)	Median (IQR)
**Gender**	Female	21.0 (12.0–33.0)	18.0 (12.0–24.0)	20.0 (12.0–26.0)	10.0 (7.0–12.0)
	Male	13.5 (5.5–25.0)	13.5 (7.0–22.0)	14.5 (8.5–21.5)	9.0 (6.0–10.0)
*p* *		**<0.001**	**0.002**	**<0.001**	**0.002**
**Marital status**	Married	23.0 (12.0–33.0)	17.0 (11.0–23.0)	20.0 (12.0–25.0)	10.0 (7.0–12.0)
	Single	15.0 (6.0–25.0)	15.0 (8.0–22.0)	15.0 (10.0–22.5)	9.0 (6.0–11.0)
*p* *		**<0.001**	0.097	**0.011**	**0.018**
**Occupation**	Doctor	7.0 (1.0–16.5) ^a^	12.5 (2.0–26.5)	7.5 (4.5–14.5) ^a^	6.5 (4.5–10.0) ^a^
	Nurse/midwife	19.5 (11.0–33.0) ^b^	17.0 (10.0–23.0)	17.0 (12.0–24.0) ^b^	9.0 (7.0–12.0) ^b^
	Technician	23.5 (14.5–30.5) ^b^	14.0 (10.0–20.0)	18.0 (11.5–26.0) ^b^	9.5 (7.0–12.0) ^b^
	Medical secretary	19.0 (7.0–27.0) ^b^	16.0 (11.0–23.0)	21.0 (15.0–26.0) ^b^	10.0 (8.0–11.0) ^b^
	Other	12.0 (5.5–20.0) ^a,b^	20.5 (10.0–22.5)	19.5 (12.0–24.5) ^b^	10.0 (8.5–11.0) ^b^
*p* **		**0.002**	0.455	**0.007**	**0.049**
**Status of having experienced the earthquakes**	Yes	20.0 (10.0–31.0)	17.0 (10.0–23.0)	18.0 (11.0–25.0)	9.0 (7.0–12.0)
	No	15.0 (4.0–22.0)	13.5 (7.0–27.0)	13.5 (6.0–17.0)	8.0 (6.0–10.0)
*p* *		0.152	0.489	**0.019**	0.060
**Location during the earthquakes**	At home	20.0 (9.0–31.0)	16.0 (10.0–22.0)	18.0 (11.5–25.0)	9.0 (7.0–12.0)
	At the hospital	16.0 (12.0–28.0)	22.0 (19.0–28.0)	15.0 (9.0–27.0)	10.0 (7.0–13.0)
*p* *		0.858	**0.040**	0.930	0.775
**Presence of damage to home**	Yes	20.0 (10.0–32.0)	17.0 (11.0–23.0)	18.0 (12.0–25.0)	10.0 (7.0–12.0)
	No	14.0 (5.0–23.0)	12.5 (6.0–18.0)	14.5 (6.0–21.0)	8.0 (6.0–10.0)
*p* *		**0.005**	**0.005**	**0.004**	**0.001**
**Current place of residence**	Own house	19.0 (10.0–28.0)	15.0 (10.0–22.0)	18.0 (11.0–25.0)	9.0 (7.0–12.0)
	Temporaryhousing	19.0 (8.0–32.0)	17.0 (10.0–23.0)	17.0 (11.0–24.0	10.0 (7.0–12.0)
*p* *		0.914	0.259	0.770	0.509
**Injury status in the earthquake**	Yes	26.0 (13.0–38.0)	21.0 (14.0–31.0)	1.5 (12.0–26.0)	11.0 (8.0–13.0)
	No	18.0 (8.0–28.0)	15.0 (9.0–22.0)	16.0 (11.0–24.0)	9.0 (7.0–11.0)
*p* *		**0.002**	**<0.001**	**0.028**	**0.004**
**Presence of injured family members**	Yes	23.5 (12.5–33.5)	19.0 (13.0–27.0)	22.0 (15.5–26.0)	10.0 (7.5–12.0)
	No	17.0 (8.0–28.0)	15.0 (8.0–22.0)	16.0 (10.0–24.0)	9.0 (7.0–12.0)
*p* *		**0.017**	**0.001**	**<0.001**	**0.036**
**Loss of relatives in the earthquake**	Yes	20.0 (11.0–33.0)	17.0 (11.0–23.0)	20.0 (13.0–26.0)	10.0 (7.0–12.0)
	No	12.5 (5.0–23.0)	14.0 (7.0–21.0)	11.0 (6.0–17.0)	7.5 (5.0–10.0)
*p* *		**0.001**	**0.035**	**<0.001**	**<0.001**

* The Mann–Whitney U test was performed, ** The Kruskal–Wallis test was performed. ^a,b^ The group from which the difference originates.

**Table 3:** Correlations of scale scores.

		BAI	BDI	PTSD	PSQI
**BDI**	r	**0.474**			
	*p*	**<0.001**			
**PTSD**	r	**0.690**	**0.430**		
	*p*	**<0.001**	**<0.001**		
**PSQI**	r	**0.507**	**0.414**	**0.579**	
	*p*	**<0.001**	**<0.001**	**<0.001**	
**Age**	r	**0.169**	0.002	0.111	**0.144**
	*p*	**0.003**	0.971	0.055	**0.013**
**Educational status**	r	**−0.173**	0.013	**−0.119**	−0.087
	*p*	**0.003**	0.827	**0.041**	0.133
**Income status**	r	−0.066	−0.085	−0.109	**−0.173**
	*p*	0.255	0.144	0.059	**0.003**
**Weekly working hours**	r	0.001	**0.142**	−0.065	−0.018
	*p*	0.992	**0.014**	0.259	0.753
**Duration of marriage**	r	0.118	−0.046	−0.005	0.013
	*p*	0.123	0.547	0.944	0.863
**Number of years in the profession**	r	**0.163**	0.014	**0.147**	**0.152**
	*p*	**0.005**	0.803	**0.011**	**0.008**
**Level of damage to home**	r	0.056	0.090	0.032	**0.137**
	*p*	0.371	0.153	0.609	**0.028**

The Spearman’s rank correlation test was performed.

**Table 4:** Linear regression analysis of scale scores.

	β	SE	Standardized β	t	*p*
BAI (R^2^ = 0.549; F = 28.869; *p* < 0.001)					
**BDI**	0.263	0.060	0.198	40.378	<0.001
**PTSD**	0.928	0.083	0.577	110.224	<0.001
**PSQI**	0.349	0.203	0.085	10.715	0.087
**Age**	0.356	0.138	0.219	20.586	0.010
**Gender**	−0.227	10.265	−0.008	−0.180	0.858
**Educational status**	−20.010	0.743	−0.112	−20.705	0.007
**Marital status**	−10.743	10.333	−0.061	−10.308	0.192
**Number of years in the profession**	−0.253	0.137	−0.153	−10.841	0.067
**Occupation**	−20.428	0.687	−0.155	−30.533	<0.001
**Presence of damage to home**	−0.890	10.635	−0.022	−0.544	0.587
**Injury status in the earthquake**	−10.395	10.612	−0.037	−0.865	0.388
**Presence of injured family members**	10.736	10.346	0.054	10.289	0.198
**Loss of relatives in the earthquake**	20.251	10.471	0.065	10.530	0.127
**BDI (R^2^ = 0.293; F = 12.617; *p* < 0.001)**					
**BAI**	0.238	0.054	0.315	40.447	<0.001
**PTSD**	0.037	0.093	0.030	0.395	0.693
**PSQI**	0.565	0.189	0.186	20.981	0.003
**Age**	−10.992	10.177	−0.090	−10.692	0.092
**Weekly working hours**	10.770	0.922	0.102	10.919	0.056
**Location during the earthquakes**	30.444	20.295	0.078	10.501	0.135
**Presence of damage to home**	−0.566	10.665	−0.017	−0.340	0.734
**Injury status in the earthquake**	−30.419	10.518	−0.124	−20.252	0.025
**Presence of injured family members**	−10.453	10.299	−0.061	−10.119	0.264
**Loss of relatives in the earthquake**	−0.408	10.446	−0.015	−0.282	0.778
**PTSD (R^2^ = 0.581; F = 30.509; *p* < 0.001)**					
**BAI**	0.325	0.029	0.522	110.044	<0.001
**BDI**	0.021	0.037	0.026	0.569	0.570
**PSQI**	0.598	0.117	0.235	50.103	<0.001
**Gender**	−20.262	0.754	−0.123	−30.001	0.003
**Marital status**	0.729	0.786	0.041	0.927	0.355
**Educational status**	0.509	0.445	0.046	10.144	0.254
**Occupation**	10.488	0.413	0.153	30.605	<0.001
**Number of years in the profession**	0.009	0.046	0.009	0.197	0.844
**Status of having experienced the earthquakes**	−20.042	10.484	−0.055	−10.376	0.170
**Weekly working hours**	−0.125	0.576	−0.009	−0.217	0.828
**Presence of damage to home**	0.216	10.012	0.009	0.213	0.831
**Injury status in the earthquake**	10.109	0.977	0.047	10.136	0.257
**Presence of injured family members**	−10.849	0.805	−0.093	−20.296	0.022
**Loss of relatives in the earthquake**	−20.766	0.874	−0.128	−30.165	0.002
**PSQI (R^2^ = 0.337; F = 10.952; *p* < 0.001)**					
**BAI**	0.037	0.019	0.151	10.937	0.054
**BDI**	0.047	0.020	0.142	20.305	0.022
**PTSD**	0.132	0.031	0.334	40.283	<0.001
**Gender**	−0.174	0.423	−0.024	−0.411	0.682
**Age**	0.009	0.043	0.023	0.214	0.831
**Marital status**	0.220	0.426	0.031	0.517	0.606
**Income status**	−0.395	0.268	−0.079	−10.475	0.142
**Occupation**	0.190	0.222	0.049	0.855	0.394
**Number of years in the profession**	0.037	0.044	0.089	0.840	0.402
**Injury status in the earthquake**	−0.353	0.515	−0.039	−0.685	0.494
**Presence of injured family members**	0.302	0.437	0.039	0.691	0.490
**Loss of relatives in the earthquake**	−0,749	0,491	−0.083	−10.527	0.128
**Level of damage to home**	0.265	0.178	0.082	10.489	0.138

## Data Availability

The data that support the findings of this study are not openly available, but are available from the corresponding author upon reasonable request.
